# Evaluation of the Inhibitory Potential of Apigenin and Related Flavonoids on Various Proteins Associated with Human Diseases Using AutoDock

**DOI:** 10.3390/ijms26062548

**Published:** 2025-03-12

**Authors:** Tanat Peanlikhit, Uma Aryal, James S. Welsh, Kenneth R. Shroyer, Kanokporn Noy Rithidech

**Affiliations:** 1 Pathology Department, Stony Brook University, Stony Brook, NY 11794-8691, USA; tanat.peanlikhit@stonybrookmedicine.edu (T.P.); kenneth.shroyer@stonybrookmedicine.edu (K.R.S.); 2 Department of Comparative Pathobiology, Purdue University, West Lafayette, IN 47907, USA; uaryal@purdue.edu; 3 Purdue Proteomics Facility, Bindley Bioscience Center, Purdue University, West Lafayette, IN 47907, USA; 4 Department of Radiation Oncology, Loyola University Health System, Maywood, IL 60153, USA; james.welsh@va.gov

**Keywords:** apigenin, flavonoids, molecular docking, KRAS mutations, oxidative stress and inflammation, bacterial infections

## Abstract

We used molecular docking to determine the binding energy and interactions of apigenin and 16 related flavonoids, with 24 distinct proteins having diverse biological functions. We aimed to identify potential inhibitors of these proteins and understand the structural configurations of flavonoids impacting their binding energy. Our results demonstrate that apigenin exhibits high binding energies (a surrogate for binding affinity or inhibitory potential) to all tested proteins. The strongest binding energy was −8.21 kcal/mol for p38 mitogen-activated protein kinases, while the weakest was −5.34 kcal/mol for cyclin-dependent kinase 4. Apigenin and many other flavonoids showed high binding energies on xanthine oxidase (1.1–1.5 fold of febuxostat) and DNA methyltransferases (1.1–1.2 fold of azacytidine). We uncovered high binding energies of apigenin and certain flavonoids with mutated Kirsten rat sarcoma viral oncogene homolog at G12D (KRAS G12D), G12V, and G12C. Consequently, apigenin and certain flavonoids have the potential to effectively inhibit pan-KRAS oncogenic activity, not just on specific KRAS mutations. Apigenin and certain flavonoids also have high binding energies with aromatase (involved in estrogen production) and bacterial infections, i.e., DNA gyrase B and 3R-hydroxy acyl-ACP dehydratase (FABZ). Our findings are pivotal in identifying specific flavonoids that can effectively inhibit targeted proteins, paving the way for the development of innovative flavonoid-based drugs.

## 1. Introduction

Apigenin and other flavonoids have gained interest due to their pharmacological activities counteracting oxidative stress, inflammation, cancer, and microbial infections, including diseases of the central nervous and cardiovascular systems [1,2,3]. Importantly, apigenin and other flavonoids promote the proliferation of normal stem cells [4,5,6] while inhibiting the growth of cancer stem cells [7,8]. Furthermore, unlike many synthetic compounds, apigenin and other flavonoids have a favorable safety profile, making them an attractive option for clinical applications as single or adjunct drugs to conventional treatments, aiming to reduce the side effects and resistance to those treatments. For several years, our research has focused on apigenin, a flavone subclass of the flavonoid family. This is because apigenin has shown great potential in combating inflammation and oxidative stress induction by radiation [9,10,11,12], as well as anti-cancer and anti-microbial infection [13,14,15,16,17,18,19]. Our team was the first to discover that apigenin protects cultured human lymphocytes when given before γ-irradiation [9] and after irradiation with γ-irradiation [10], highlighting its medical countermeasure (MCM) capabilities. Other researchers have since confirmed our findings on the beneficial effects of apigenin in both in vitro [20,21,22,23] and in vivo [11,12,24] systems. Recently, we discovered that apigenin given as a diet supplement to mice before and after exposure to space-like radiation prevents leukopenia and thrombocytopenia, reduces the frequencies of radiation-induced chromosome aberrations, increases erythropoiesis and the proliferation of hematopoietic stem/progenitor cells, including the suppression of the activation of nuclear factor-kappa B (NF-κB) and inflammation in bone marrow (BM) cells [6] and in the duodenum [25], as well as restores the balance of the gut microbiota [25]. It is also important to note that apigenin has a favorable safety profile [26,27]. No acute toxicity or mortality was reported in mice or rats given apigenin at oral doses up to 5000 mg/kg bw [28]. Hence, the remarkable beneficial effects and favorable safety profile of apigenin make it an excellent flavonoid for developing as a therapeutic drug to be used alone or combined with other flavonoids, as well as an adjunct therapy with conventional drugs.

Molecular docking has become a crucial component of in silico drug development [29]. This computer-aided drug discovery method [30,31] is used to determine the binding energy in kcal/mol and the interaction of a ligand (drug) molecule, such as the flavonoid, with a pocket (usually the active or functional site) of a receptor (e.g., a protein). Hence, molecular docking enables the study of the structure–function relationships of the ligand and the receptor. In drug design, inhibitory potential of tested compounds are typically required to have a negative value binding energy equal to or higher than −6.0 kcal/mol [32]. However, binding energy with a negative value about −5 kcal/mol has been considered a suitable inhibitor by the FDA to approve some drugs against HIV-1 reverse transcriptase, e.g., −5.63 kcal/mol for abacavir (ABC), −5.68 kcal/mol for zidovudine (AZT) [33]. Notably, the greater the negative binding energy released during docking, the more stable the resulting complex is likely to be, which is associated with the high binding affinity resulting in a high inhibitory potential. To date, popular and freely available molecular docking software, such as AutoDock 4.2.6 [34] and AutoDock Vina 1.2 [35], can be used for this purpose. While AutoDock Vina is faster and provides more accurate binding poses, AutoDock 4.2.6 has better performance in terms of the predicted binding affinity with experimental values [36].

The primary objective of our study was to identify potential inhibitors for 24 selected proteins (or receptors) and to understand how the structural configurations of flavonoids influence their binding energy (binding affinity or inhibitory effects). To achieve this goal, we utilized the AutoDock 4.2.6 molecular docking software to analyze the binding energy values and interactions of apigenin and 16 other flavonoids (details are provided in Table S1) with the 24 different proteins (details are provided in Table S2) known to be associated with oxidative stress, inflammation, carcinogenesis, and bacterial infections. We also included drugs that are known to inhibit each receptor as positive controls, allowing us to directly compare binding energy and inhibition constant (Ki) values. The binding energies obtained from our study are essential for future drug development involving flavonoids, as they will help in selecting the most effective interactions between flavonoids and their target proteins to enhance pharmacological activity.

## 2. Results

We used the three-dimensional crystal structures of receptors to identify amino acids located in the active site for docking purposes. From the Protein Data Bank (PDB_ID) database, we obtained 35 three-dimensional crystal structures of proteins among the 24 receptors, which served as the active sites (pockets) for docking. Detailed information for each PDB_ID is presented in Table S3 of the Supplemental Data. For each receptor, we present the binding energies (in kcal/mol) and Ki constants (in µM) derived from AutoDock 4.2.6, along with 3D images that illustrate the interactions between the ligands and the receptors. It is important to note that the inhibition constant (Ki) is inversely related to the binding energy of the ligand–receptor complex. Using Chimera X 1.5 software, 3D images showed predicted hydrogen bonds (dotted blue lines) between ligands and amino acids within the active sites of each receptor. In these representations, oxygen atoms are colored red, hydrogen atoms are shown in white, and nitrogen atoms are dark blue. To distinguish between different classes of compounds, apigenin is represented in green, genistein in pink, and quercetin in yellow, corresponding to flavones, isoflavones, and flavonols, respectively. The notation “na” in each illustration indicates “not applicable”. Further details of our results are presented below.

### 2.1. Binding Energy of Apigenin and Related Compounds with Proteins Known to Be Involved in Oxidative Stress

**Figure 1:**
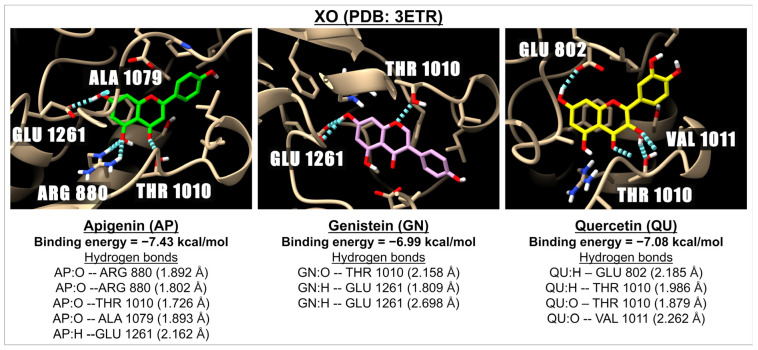
The 3D images illustrate the interactions between three compounds: apigenin (green), genistein (pink), and quercetin (yellow) with a receptor involved in oxidative stress, specifically XO (PDB: 3ETR). The images highlight hydrogen bonds (represented by dotted blue lines) formed between the ligands (with oxygen molecules shown in red and hydrogen molecules in white) and the amino acids located in the active site of the XO receptor.

Table 1 presents the binding energies and inhibition constants (Ki) of apigenin and related flavonoids with NOX, XO, and iNOS. The results indicate that apigenin and baicalin have the highest binding energies to NOX, with values of −6.83 and −7.16 kcal/mol, respectively. Among the compounds tested, flavones are the most effective in inhibiting the activity of the NOX enzymes. Setanaxib, a NOX inhibitor currently in Phase 2 clinical trials, demonstrated a higher binding energy to NOX than the tested flavonoids, measuring at −8.35 kcal/mol; however, its safety is still under evaluation. For the XO enzyme, two active sites were analyzed: PDB: 1FIQ and PDB: 3ETR. The binding energy results reveal that all tested flavonoids effectively inhibit the activity of the XO enzymes at both active sites, with binding energies ranging from −6.33 kcal/mol (baicalin) to −8.77 kcal/mol (fisetin) for XO (PDB: 1FIQ), and from −6.12 kcal/mol (vitexin) to −8.70 kcal/mol (fisetin) for XO (PDB: 3ETR). Most of the tested flavonoids against XO (PDB: 1FIQ) are more effective than the known XO inhibitor febuxostat, which has a binding energy of −6.94 kcal/mol. Notably, the flavonoids also surpassed febuxostat in effectiveness against XO (PDB: 3ETR). Regarding iNOS, apigenin and other tested flavones, including genistein and daidzein (isoflavones), showed greater binding effectiveness than flavonols. Figure 1 illustrates the 3D representations of the interactions between apigenin, genistein, and quercetin with XO (PDB: 3ETR).

### 2.2. Binding Energies of Apigenin and Related Compounds with Proteins Known to Be Involved in Inflammation

**Figure 2:**
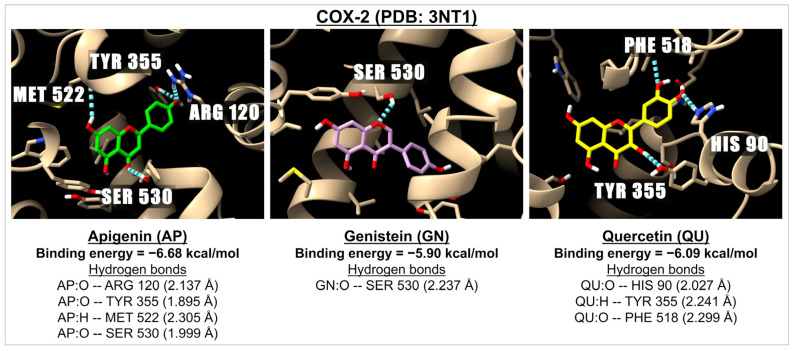
The 3D images illustrate the interactions between apigenin (in green), genistein (in pink), and quercetin (in yellow) with a receptor involved in inflammation, i.e., COX-2 (PDB: 3NT1). The images highlight hydrogen bonds formed between the ligands (represented by the red oxygen molecules and white hydrogen molecules) and the amino acids located in the active site of the COX-2 receptor.

Our data, as shown in Table 2, indicate that there is strong binding energy between apigenin and selected flavonoids with IKK, ranging from −6.44 kcal/mol (myricetin) to −7.98 kcal/mol (baicalin). Among the aglycone flavones (apigenin, luteolin, chrysin, and baicalein), apigenin exhibits the highest binding energy with IKK at −7.45 kcal/mol. Furthermore, our results demonstrate that glycoside and methoxide flavones form better complexes with IKK compared to those in aglycone form. Currently, IKK does not have any known inhibitors. All tested flavonoids form complexes with p38 MAPK, resulting in high binding energies that range from −6.85 kcal/mol (isoorientin) to −9.91 kcal/mol (baicalin). We used adezmapimod, a known p38 MAPK inhibitor, as a positive control, which yielded a binding energy of −9.96 kcal/mol, comparable to that of baicalin. The p38 MAPK plays a crucial role in inflammation and regulates the activation of NF-κB, a key transcription factor that controls the expression of many genes involved in inflammatory responses and cell survival. We investigated both heterodimers of NF-κB, specifically p50–p65 NF-κB (the most common heterodimer associated with the canonical NF-κB activation) and p52–p65 NF-κB (which results from non-canonical NF-κB activation). The data show that apigenin and selected flavonoids (excluding baicalin and myricetin, which have a binding energy of −4.72 kcal/mol) are good ligands for the p50–p65 NF-κB heterodimer, while all studied flavonoids serve as good ligands for the p52–p65 NF-κB heterodimer. Sulfasalazine, an NF-κB inhibitor, is more effective at inhibiting both NF-κB heterodimers than apigenin and the tested flavonoids. Additionally, apigenin and selected flavonoids act as good ligands for the COX-2 enzyme, with binding energies ranging from −5.29 kcal/mol (myricetin) to −7.00 kcal/mol (vitexin). By comparison, the binding energy of COX-2 with naproxen, a non-selective nonsteroidal anti-inflammatory drug (NSAID) used as a COX-2 inhibitor, is −7.61 kcal/mol. Examples of 3D images illustrating the interactions between apigenin, genistein, and quercetin with COX-2 (PDB: 3NT1) can be found in Figure 2.

### 2.3. Binding Energy of Apigenin and Related Compounds with Proteins Known to Be Involved in Carcinogenesis

Table 3 presents the binding energies and inhibition constants (Ki) of apigenin and related compounds for mutated EGFR and mutated KRAS. We examined the interactions with the mutated EGFR variant T790M/V948R (PDB: 6Z4B). The data indicate that apigenin and the tested flavonoids exhibit strong binding to EGFR T790M/V948R, with binding energies ranging from −6.32 kcal/mol (for tricin) to −8.08 kcal/mol (for baicalin). However, these binding energies are lower than those of established EGFR inhibitors, such as erlotinib and gefitinib, which have binding energies of −8.86 and −8.20 kcal/mol, respectively.

Additionally, we assessed the binding energy and Ki values of apigenin and related flavonoids against three types of mutated KRAS (the most commonly oncogenic proteins found in solid tumors [37,38]), specifically KRAS G12C, KRAS G12D, and KRAS G12V. We configured the grids to cover the switch I and switch II regions separately. For KRAS G12C (PDB: 4LV6), the binding energies for switch I ranged from −6.25 kcal/mol (for hispidulin) to −7.87 kcal/mol (for orientin), while for switch II, they ranged from −6.03 kcal/mol (for hispidulin) to −8.28 kcal/mol (for baicalin). These binding energies are lower than those of sotorasib and adagrasib, the two FDA-approved inhibitors for KRAS G12C.

For KRAS G12D (PDB: 6GJ8), the binding energies with switch I of various flavonoids were found to be higher than that of MRTX1133 (a synthetic small molecule known to suppress the oncogenic activity of the KRAS G12D [39], with a binding energy of −7.67 kcal/mol. The binding energies of flavonoids higher than that of MRTX1133 were as follows: apigenin (−7.86 kcal/mol), baicalein (−7.94 kcal/mol), isovitexin (−7.89 kcal/mol), hispidulin (−7.76 kcal/mol), genistein (−8.83 kcal/mol), daidzein (−8.66 kcal/mol), kaempferol (−7.71 kcal/mol), fisetin (−7.85 kcal/mol), and myricetin (−7.94 kcal/mol). Nonetheless, all tested flavonoids exhibited low to moderate inhibitory potency for switch II of KRAS G12D, with the binding energies ranging from –4.49 (myricetin) to −5.74 (chrysin) kcal/mol, which were lower than that of MRTX1133 (−7.49 kcal/mol).

**Figure 3:**
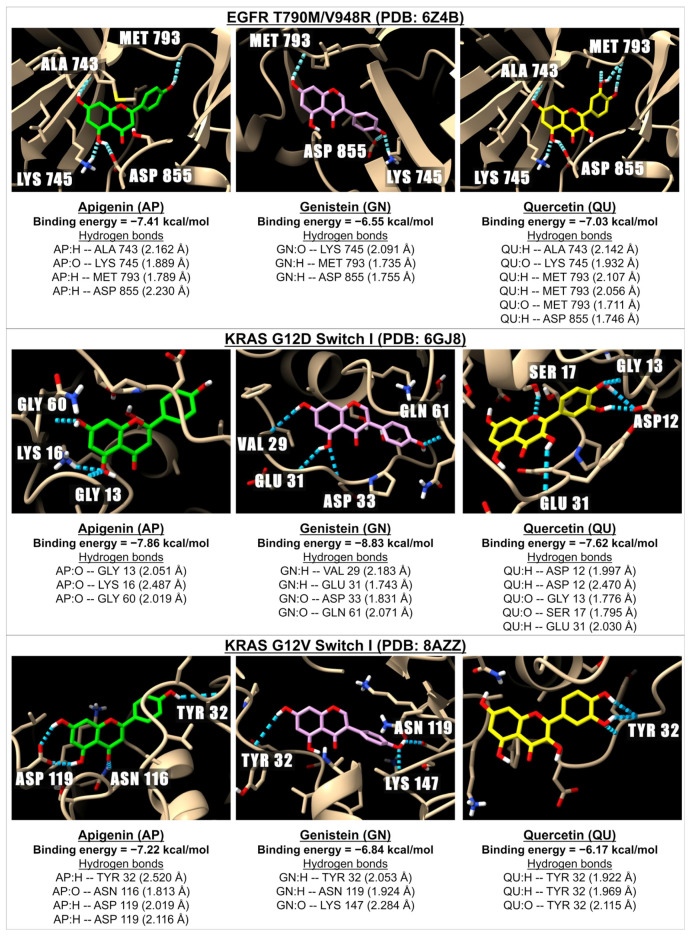
The 3D images illustrate the interactions between apigenin (in green), genistein (pink), and quercetin (yellow) with receptors involved in EGFR and KRAS pathways: EGFR T790M/V948R (PDB: 6Z4B), KRAS G12D (PDB: 6GJ8), and KRAS G12V (PDB: 8AZZ). The images depict hydrogen bonds (dotted blue lines) between the ligand (oxygen molecules in red and hydrogen molecules in white) and the amino acids located in EGFR and KRAS.

For KRAS G12V (PDB: 8AZZ), the binding energies of switch I with apigenin, chrysin, and baicalein were −7.22 kcal/mol, −7.44 kcal/mol, and −7.34 kcal/mol, respectively, exceeding that of BI-2865 (a synthetic drug known for inhibiting the activation of KRAS G12V), which had binding energy of −6.97 kcal/mol. The binding energies for switch II of KRAS G12V ranged from −5.90 kcal/mol for tricin to −7.95 kcal/mol for genistein, both of which were lower than that of BI-2865, which exhibited a binding energy of −11.12 kcal/mol. Figure 3 presents 3D images illustrating the interactions and binding energies between apigenin, genistein, and quercetin with a receptor involved in EGFR, as well as the switch I of KRAS mutations, specifically EGFR T790M/V948R (PDB: 6Z4B), KRAS G12D (PDB: 6GJ8), and KRAS G12V (PDB: 8AZZ).

Table 4 shows the binding energies and inhibition constants (Ki) between apigenin and related flavonoids with the RAF/MEK/ERK signaling pathway, which is one of the downstream pathways regulated by the activation of KRAS. For the BRAF proteins, we investigated the complexes of apigenin and related compounds with BRAF-mutated V599E (PDB: 1UWJ) and BRAF-mutated V600E (PDB: 3OG7). Our results show that apigenin and all tested flavonoids are efficiently bound to the pocket sites of BRAF, particularly with BRAF V599E (PDB: 1UWJ) with binding energies ranging from −6.43 (quercetin) to −9.23 (baicalin) kcal/mol; while binding energies ranged from −5.05 (myricetin) to −7.72 (baicalin) kcal/mol for BRAF V600E (PDB: 3OG7). Further, our results indicate that the flavones are better ligands for these pockets of the RAF proteins than those of isoflavones and flavonols. Nonetheless, the binding energies of the currently known RAF inhibitors with these mutated BRAF are higher (i.e., encorafenib and dabrafenib, ranging from −7.85 to −13.27 kcal/mol) than those of apigenin and selected flavonoids.

**Figure 4:**
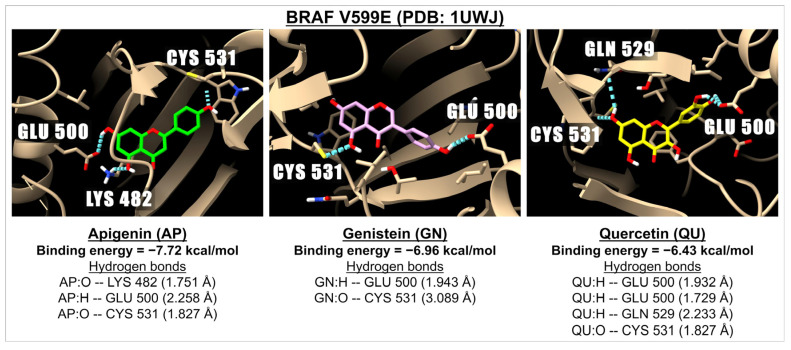
The 3D images illustrate the interaction and binding energies of apigenin (green), genistein (pink), and quercetin (yellow) with the BRAF V599E (PDB: 1UWJ) which is involved in the RAF/MEK/ERK signaling pathway. The images depict hydrogen bonds (represented by dotted blue lines) between the ligand (with oxygen molecules in red and hydrogen molecules in white) and amino acids located in the BRAF.

As for the binding energy and Ki values of apigenin and related compounds with MEK-1 C121S (PDB: 7F2X) and ERK-2 G169D (PDB: 6D5Y), our results show that all flavonoids are effective in binding to MEK-1 C121S (PDB: 7F2X), with binding energies ranging from −5.74 (myricetin) to −7.07 (tricin) kcal/mol. The binding energy of apigenin with MEK-1 C121S (PDB: 7F2X) was −6.58 kcal/mol, which is not so different from that of selumetinib (−6.81 kcal/mol), an FDA-approved MEK-1 inhibitor [40], but less potent than trametinib (−8.96 kcal/mol), which is another FDA-approved inhibitor for the MEK-1 drug [41]. Further, apigenin and other flavonoids selected for study are good ligands for binding with ERK-2 G169D (PDB: 6D5Y) resulting in a binding energy ranging from −5.14 (myricetin) to −6.41 (baicalin) kcal/mol, while the binding energy of ulixertinib (the FDA-approved ERK-2 inhibitor) is −7.85 kcal/mol. The 3D images illustrating the interaction between BRAF V599E (PDB: 1UWJ) and apigenin, genistein, or quercetin are presented in Figure 4.

**Figure 5:**
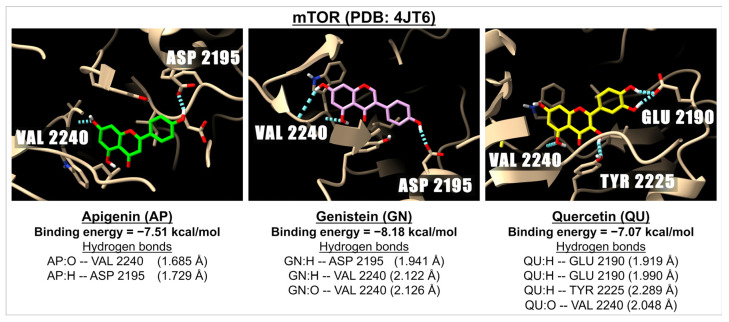
The 3D images show examples of the interaction of the molecular docking and binding energies between apigenin (green), genistein (pink), and quercetin (yellow) with a receptor involved in the PI3K, AKT, and mTOR pathway i.e., mTOR (PDB: 4JT6). The images show hydrogen bonds (dotted blue lines) between the ligand (oxygen molecules in red and hydrogen molecules in white) and amino acids in the mTOR active sites.

Table 5 presents the binding energies and inhibition constants (Ki) of apigenin and related flavonoids with the receptors of the PI3K/AKT/mTOR signaling pathway. The results indicate that apigenin and the related flavonoids can effectively bind to PI3K (PDB: 1E7U), AKT (PDB: 3O96), and mTOR (PDB: 4JT6). The binding energies range from −5.39 kcal/mol (isoorientin) to −7.37 kcal/mol (tricin) for PI3K, from −6.03 kcal/mol (myricetin) to −8.64 kcal/mol (vitexin) for AKT, and from −6.88 kcal/mol (myricetin) to −8.61 kcal/mol (baicalin) for mTOR. These findings demonstrate that apigenin and all the selected flavonoids exhibit strong inhibitory activities against the PI3K/AKT/mTOR pathway. Among the tested flavonoids, baicalin shows the highest binding energies for this pathway, with values of −7.32 kcal/mol for PI3K, −8.27 kcal/mol for AKT, and −8.61 kcal/mol for mTOR. Notably, the binding energy of baicalin with PI3K is comparable to that of the FDA-approved PI3K inhibitor buparlisib, which has a binding energy of −7.16 kcal/mol. However, the binding energies of baicalin with AKT and mTOR are lower than those of known AKT inhibitors, which are −9.73 kcal/mol for GSK690693 (an AKT inhibitor) and −9.22 kcal/mol for PI-103 (an mTOR inhibitor). Figure 5 shows the 3D images of the docking between mTOR (PDB: 4JT6) and apigenin, genistein, or quercetin are presented as examples of the interaction between a ligand and a receptor of this pathway.

**Figure 6:**
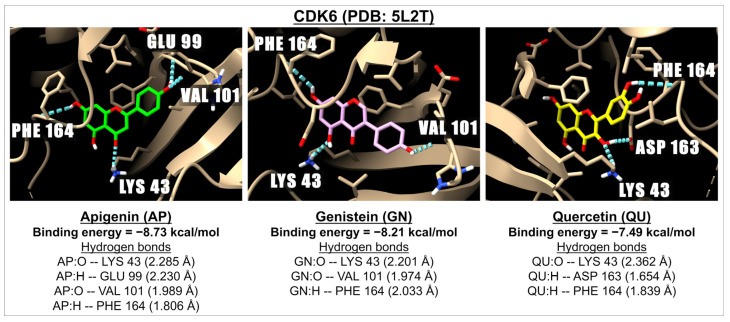
The 3D images illustrate the interaction between the molecular docking and binding energies of three compounds: apigenin (green), genistein (pink), and quercetin (yellow) with the cyclin-dependent kinases 6 (CDK6) receptor (PDB: 5L2T). The images highlight the hydrogen bonds (represented by dotted blue lines) between the ligand (with oxygen molecules in red and hydrogen molecules in white) and amino acids within one of the active sites of CDK6.

Table 6 presents the binding energies and inhibition constants (Ki) of apigenin and related flavonoids with CDK2 (three active sites), CDK4 (two active sites), and CDK6 (three active sites). Our data indicate that apigenin and related flavonoids serve as effective ligands for all three types of CDK proteins, with a particular strength of interaction observed with CDK6 (PDB: 1XO2), showing binding energies ranging from −7.62 kcal/mol (tricin) to −9.35 kcal/mol (orientin). For CDK2 (PDB: 5L2T), the binding energies range from −6.92 kcal/mol (orientin) to −9.77 kcal/mol (isovitexin). Notably, these binding energies are lower than those of synthetic drugs known to inhibit the activities of CDK2 and CDK6. Figure 6 shows 3D images of the docking interactions between CDK6 (PDB: 5L2T) and apigenin, genistein, and quercetin, serving as examples of the interactions between these ligands and their receptor in this pathway.

**Figure 7:**
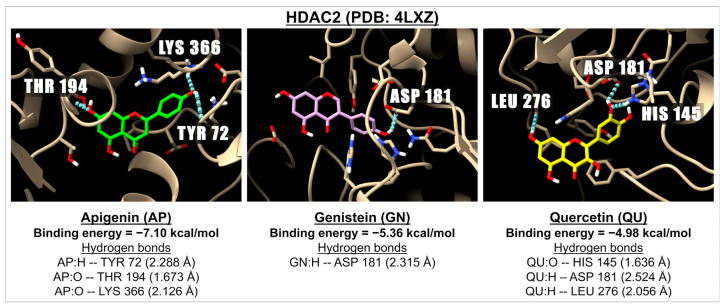
The 3D images illustrate the interaction of the molecular docking and binding energies between three compounds: apigenin (green), genistein (pink), and quercetin (yellow) with a receptor involved in epigenetics, specifically HDAC2 (PDB: 4LXZ). The images show hydrogen bonds, depicted as dotted blue lines between the ligand (oxygen molecules in red and hydrogen molecules in white) and amino acids within one of the active sites of HDAC2.

Table 7 presents the binding energies and inhibition constants (Ki) of apigenin and related flavonoids with the aromatase inhibitor, DNMT1, HDAC1, and HDAC2. We examined the two known active sites of aromatase: PDB: 3EQM and PDB: 5JKV. Our findings indicate that most flavonoids are effective ligands for aromatase (PDB: 3EQM), with binding energies ranging from −5.31 kcal/mol (myricetin) to −7.53 kcal/mol (baicalin). Additionally, apigenin and related flavonoids demonstrate a stronger binding affinity for PDB: 3EQM compared to PDB: 5JKV. Notably, letrozole, a well-known aromatase inhibitor, forms a complex with aromatase, exhibiting a binding energy of −7.77 kcal/mol at both active sites. Interestingly, apigenin and related flavonoids show significant inhibition activity against DNMT1 (PDB: 3AV6), with binding energies varying from −6.26 kcal/mol (isoorientin) to −8.49 kcal/mol (baicalin). Among the flavonoids tested, nine (apigenin, luteolin, baicalin, vitexin, hispidulin, tricin, quercetin, kaempferol, and fisetin) exhibit greater potency than azacytidine, an FDA-approved DNMT1 inhibitor, which has a binding energy of −7.41 kcal/mol. For HDAC1 (PDB: 4BKX), flavones (excluding isovitexin and isoorientin) serve as good ligands, while isoflavones and flavonols are less effective compared to flavones. Notably, apigenin is identified as the most potent inhibitor for HDAC2, while isovitexin, orientin, isoorientin, and quercetin show the least effectiveness in binding to HDAC2 (PDB: 4LXZ), with binding energies ranging from −3.93 to −4.98 kcal/mol. Figure 7 illustrates 3D images of docking interactions between HDAC2 (PDB: 4LXZ) and ligands, such as apigenin, genistein, and quercetin, highlighting examples of the interactions between ligands and receptors in this pathway.

### 2.4. Binding Energies and Inhibition Constants (Ki) of Apigenin and Related Compounds with Proteins Known to Be Involved in Bacterial Infection

**Figure 8:**
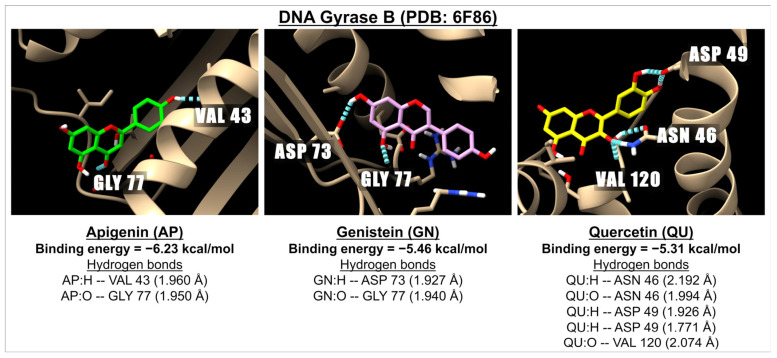
The 3D images illustrate the interaction of the molecular docking and binding energies between apigenin (green), genistein (pink), and quercetin (yellow) with a receptor involved in bacterial infection, specifically DNA Gyrase B (PDB: 6F86). The images highlight hydrogen bonds, represented by dotted blue lines, between the ligands (with oxygen molecules in red and hydrogen molecules in white) and amino acids located in one of the active sites of Gyrase B.

Table 8 presents the binding energies and inhibition constants (Ki) of apigenin and related flavonoids with DNA gyrase B (PDB: 6F86 and PDB: 4PRV) as well as FABZ (PDB: 3CF9) at sites A and B. Our findings indicate that apigenin and other flavonoids can bind to DNA gyrase at both sites, although they demonstrate a stronger binding affinity at the PDB: 4PRV site compared to PDB: 6F86. However, the well-known antibiotic ciprofloxacin exhibits greater potency in inhibiting DNA gyrase than apigenin and the tested flavonoids. In the case of FABZ, apigenin and related flavonoids show high binding energies at both active sites of PDB: 3CF9, with a stronger binding observed at site B compared to site A. Nonetheless, ciprofloxacin demonstrates a higher affinity for both active sites of FABZ (PDB: 3CF9). Figure 8 illustrates 3D images of the docking interactions between DNA gyrase B (PDB: 6F86) and apigenin, genistein, or quercetin, providing examples of the interactions between these ligands and the receptor within this pathway.

## 3. Discussion

In recent years, there have been numerous reports on using molecular docking to predict the binding energy and interaction between certain flavonoids and specific protein targets. However, these studies have focused either on one or a few flavonoids, such as apigenin, luteolin, genistein, or quercetin, and only a limited number of targeted proteins [42,43,44,45,46,47,48,49,50]. By contrast, our study compared the binding energy values and interactions of apigenin with 24 different receptors (proteins) to those of 17 flavonoids belonging to flavones, isoflavones, and flavonols. The results from our docking study using the AutoDock 4.2.6 software have produced several promising leads for future comprehensive studies on the inhibitory effects of apigenin and selected flavonoids. The binding energies obtained from our study are crucial for future flavonoid-based drug development to select the best option of interaction between flavonoids and their target proteins for better pharmacological activity. Our results unequivocally demonstrate that both apigenin (−6.83 kcal/mol) and baicalin (−7.16 kcal/mol) are highly effective flavonoids for inhibiting the NOX enzyme. It is important to note that, although their binding with NOX was not as robust as that of setanaxib (−8.35 kcal/mol), an FDA-approved NOX inhibitor, our findings still underscore their considerable potential. Notably, while baicalin has been found to exhibit renal toxicity [51], there are currently no reports of apigenin toxicity [27], while the safety of setanaxib is still under scrutiny, owing to potential liver toxicity and joint pain side effects [52]. To mitigate such side effects, an investigation into combining apigenin with setanaxib is warranted. Further, our results suggest that apigenin and other flavonoids are more effective than febuxostat, an XO inhibitor. This could greatly impact the development of new XO inhibitors, particularly those utilizing apigenin, given the side effects associated with febuxostat [53]. Our results align with previous research, showing that glycosylated flavonoids, like baicalin, have low inhibitory potency for XO, confirming that binding energy is a reliable indicator of a compound’s inhibitory potency and pharmacological activity. Our findings show that the methylation modification of flavonoids does not compromise the binding energy. Further, having more OH groups does not necessarily mean that the flavonoid will have a high affinity or more potent anti-oxidant. For instance, molecules with fewer OH groups, such as apigenin, show higher binding energies with NOX or XO compared to luteolin, quercetin, or myricetin, which have more OH groups. The position of OH groups and amino acids in the active site of the receptor may be critical to the inhibitory activities of flavonoids. However, it has been reported that the OH group on the 3-position of the C ring (flavonols’ structural characteristics) can cause DNA damage in normal cells [54,55,56], while this activity of flavonols, particularly quercetin and myricetin, may have therapeutic potential in cancer cells [57,58]. Overall, our findings are consistent with the previous studies suggesting that flavones are better ligands for the receptors involved in oxidative damage than flavonols or isoflavones [59]. Likewise, our docking results support the previous findings that flavones are more potent anti-inflammatory agents than other sub-families of flavonoids [60]. This is because the flavones included in this study show higher binding energies with the receptors involved in inflammation (i.e., IKK and p38 MAPK) than isoflavones or flavonols. Both IKK and p38 MAPK regulate the activation of NF-κB, a transcription factor controlling the expression of many genes related to inflammation or survival [61,62]. Inhibiting IKK and p38 MAPK can reduce NF-κB activation, leading to the attenuation of inflammation.

Our focus on cancer was on several pathways including EGFR/KRAS signaling, cyclin-dependent kinases, aromatase hormonal treatment therapy, and epigenetics involving DNMT and HDAC. KRAS mutations have been a therapeutic target for lung, colorectal, and pancreatic cancer for decades [63]. In this study, we specifically looked at KRAS G12C, KRAS G12D, and KRAS G12V mutants because mutations at codon 12 of the KRAS gene are the most commonly found in many types of human tumors. Our research investigated the binding energy of apigenin and selected flavonoids to the regions of switch I and switch II, which are dynamic allosteric binding pockets where RAS proteins switch between an inactive (non-oncogenic) state, when bound to guanosine diphosphate (GDP), and an active (oncogenic) state, when bound to guanosine triphosphate (GTP) [64,65]. This process is facilitated by the binding of guanine nucleotide exchange factors (GEFs), such as Son of Sevenless (SOS1), to the switch regions [66]. Therefore, apigenin, flavonoids, or drugs that bind to these switch regions would prevent the binding of GEFs to the switch regions, inhibiting the oncogenic activation of mutated KRAS proteins.

Our results have shown that apigenin and tested flavonoids are excellent ligands for KRAS G12C. Although their binding energies are not as high as those of FDA-approved KRAS G12C inhibitors (sotorasib and adagrasib), combined therapies of apigenin and tested flavonoids with the aforementioned inhibitors may reduce side effects and resistance to treatment that have been recently reported in lung and colorectal cancer patients treated with sotorasib or adagrasib [67]. For the KRAS G12D mutation, our data from AutoDock 4.2.6 suggest that apigenin and certain flavonoids have the potential to be effective inhibitors for the mutated KRAS G12D because they have higher binding energies with switch I (but not with switch II) than MRTX1133 (a known KRAS G12D inhibitor). Furthermore, our results strongly indicate that apigenin and specific flavonoids have high binding energies with the KRAS G12V mutant, suggesting their potential to inhibit pan-KRAS mutations at codon 12. These findings present a substantial advantage over drugs targeting specific mutations and signify a promising avenue for the development of effective cancer treatments. Our research has also shown that apigenin and related flavonoids demonstrate a high efficiency in binding to the downstream signaling pathways of KRAS, including the RAF/MEK/ERK and the PI3K/AKT/mTOR pathways.

Our results demonstrate that apigenin and specific flavonoids form a potent bond with the CDK6 protein (but less potent than ribociclib, a CDK6 inhibitor), presenting a potential benefit when combined with ribociclib to reduce neutropenia and gastrointestinal toxicity induced by ribociclib [68]. Similarly, a combined apigenin/specific flavonoid with letrozole (an aromatase inhibitor) may mitigate some of the serious side effects of letrozole (e.g., joint pain and nephrotoxicity) [69,70] and benefit breast cancer patients with ER+. Likewise, further studies are warranted to investigate the efficacy of apigenin and specific flavonoids combined with ciprofloxacin to reduce antibiotic resistance. Importantly, apigenin and many flavonoids bind better to the DNMT1 enzyme than azacytidine, suggesting the potential for new drugs with minimal side effects.

In summary, in our docking study, we found that apigenin and some flavonoids effectively inhibit all 24 proteins examined. Flavones demonstrated better results than isoflavones and flavonols. Apigenin and some flavones exhibited stronger binding energy than a few FDA-approved drugs in a ligand–receptor complex with a specific receptor. However, our study has certain limitations, such as the availability of protein sequences in the Protein Data Bank (PDB) critical for the results of the docking analysis, which is based on grid-based energy calculation. Further, docking provides only a static view of the potential binding conformation; while, in reality, the binding event is dynamic. Hence, in future studies, additional molecular docking software and molecular dynamics simulations [71] can be added. Despite these limitations, apigenin and certain flavonoids have the potential to serve as effective therapeutic agents. Recently, we conducted pilot studies to explore the therapeutic and radio-sensitizing effects of apigenin on pancreatic ductal adenocarcinoma (PDAC) cells carrying the KRAS G12D mutation, specifically PANC-1 cells. Our findings indicate that apigenin significantly reduced the proliferation of PDAC cells. Furthermore, we observed that apigenin enhanced the cell-killing effects of radiation on PDAC cells, highlighting its radio-sensitizing properties. It is plausible to speculate that apigenin binds to the switch regions of the KRAS G12D (as suggested in Table 3). This binding may deactivate the oncogenic activity of KRAS by hindering the binding of GEF molecules to the switch regions and reducing the levels of activated NF-κB, ultimately making cancer cells more susceptible to radiotherapy. Although the exact mechanisms underlying the therapeutic and radio-sensitizing potential of apigenin on PDAC cells remain unknown, further research into the use of apigenin and other flavonoids as adjuvant therapy for cancer treatment or other diseases should be encouraged. The current challenge remains that the poor bioavailability of flavonoids limits their clinical applications. However, innovative solutions, such as the development of nanocarriers, are actively being pursued in our and other laboratories to overcome this barrier.

## 4. Materials and Methods

### 4.1. Selection of Ligands (Flavonoids)

Seventeen flavonoids were selected for this study. These include 11 flavones, 2 isoflavones, and 4 flavonols. The chemical structure, number, and position of hydroxyl groups of each compound are shown in Table S1 (Supplemental Data). Four flavones are in the aglycone form (i.e., apigenin, luteolin, chrysin, and baicalein), five are in the glycoside form (i.e., baicalin, vitexin, isovitexin, orientin, and isoorientin), and two are in the methoxide form (i.e., hispidulin and tricin). The isoflavones and flavonols are in the aglycone form. The reason for studying the molecular docking of these flavonoids is their similarity to apigenin and their biological functions have been frequently investigated [60,72,73]. Flavones dominated our study as they possess the most anti-inflammatory [60] and anti-oxidative stress activities [59], as compared to other sub-families of flavonoids

### 4.2. Ligand Preparation

We downloaded the 3D structures of seventeen ligands listed in Table S1 from PubChem NCBI as a .sdf file. We added hydrogen atoms to the ligands and optimized their geometry structure using Avogadro software 1.2 [74], saved as a .mol2 file. The number of torsional degrees of freedom was determined by the number of rotatable bonds in the molecule, including the hydroxyl bonds and the C-C single bond between the C and B rings. Before docking, we converted all ligands to a .pdbqt file.

### 4.3. Receptor Preparation

Our study included a total of 24 receptors (proteins), listed in Table S2 along with their functions. We divided the selected receptors into four groups: oxidative stress, inflammation, carcinogenesis, and bacterial infection. We tested the binding energy (affinity) on more than one active (pocket) site in some proteins and compared the binding energy of these flavonoids to FDA-approved inhibitors or those under clinical trials (with one exception, i.e., IKK, since currently there is no information on its inhibitor). We retrieved 35 three-dimensional crystal structures of proteins from the Protein Data Bank (PDB) database in PyMOL software 2.5.4; the details are listed in Table S3. We removed water molecules, added polar hydrogen atoms and Kollman charges, assigned atom types for AutoDock 4, and saved protein structures as a .pdbqt file using ADT 4.2. The active sites of proteins were obtained from previously published articles (Table S3). We created grid box parameters to cover the binding pocket, and the grid spacing was set at 0.375 Å. The grid map type (A C HD N NA OA SA) was selected, and the AutoGrid script was used to convert grid parameters to the grid log file for the molecular docking step. Additional grid maps were created for the drugs containing fluorene (F), chlorine (Cl), sulfur (S), bromine (Br), or iodine (I) that are known to be the inhibitors for specific receptors (Table S4).

### 4.4. Molecular Docking

Molecular docking (flexible ligand with a rigid receptor) was performed by AutoDock 4.2.6 [34]. The Lamarckian genetic algorithm [75] was used for the conformation search. The number of evaluations was set at 25 (other genetic algorithm parameters are shown in Figure S1). All docking parameters were saved as a .dpf file. The best predicted conformation (lowest estimated free energy of binding) for each ligand and each receptor was selected and used for molecular docking visualization. The free energy of binding was calculated by:

Estimated Free Energy of Binding = Final Intermolecular Energy (Van de waals + Hydrogen bond + desolv Energy) + (Final Total Internal Energy) + (Torsional Free Energy) − (Unbound System’s Energy)

As previously mentioned, the binding energy was presented as kcal/mol, and a ligand with a binding energy of a higher negative value than −6 kcal/mol is considered a suitable inhibitor. The inhibition constants (Ki) for each receptor–ligand interaction was also calculated by AutoDock 4.2.6 and given as µM. The receptor–ligand complex was generated in ADT 4.2 and saved as a .pdbqt file. The 3D conformation graphic and predicted hydrogen bonds were generated using Chimera X 1.5 software [76]. We provided examples of the receptor–ligand interactions for each category of ligands in Figure 1, Figure 2, Figure 3, Figure 4, Figure 5, Figure 6, Figure 7 and Figure 8.

## Figures and Tables

**Table 1:** Binding energies and inhibition constants (Ki) of apigenin and related compounds with NOX, XO, and iNOS obtained from AutoDock 4.2.6.

Sub- Family	Flavonoids	NOX PDB: 2CDU		XO PDB: 1FIQ		XO PDB: 3ETR		iNOS PDB: 4NOS	
		Binding Energy (kcal/mol)	Ki (µM)	Binding Energy (kcal/mol)	Ki (µM)	Binding Energy (kcal/mol)	Ki (µM)	Binding Energy (kcal/mol)	Ki (µM)
Flavones	Apigenin	−6.83	9.90	−7.74	2.12	−7.43	3.56	−7.05	6.85
	Luteolin	−6.21	27.88	−7.54	2.99	−6.92	8.50	−5.87	49.73
	Chrysin	−6.35	22.17	−8.23	0.92	−8.05	1.25	−7.03	7.08
	Baicalein	−6.07	35.56	−8.48	0.61	−8.25	0.90	−6.98	7.62
	Baicalin	−7.16	5.68	−6.33	23.03	−6.27	25.44	−7.30	4.47
	Vitexin	−6.23	26.95	−6.96	7.95	−6.12	32.86	−6.64	13.63
	Isovitexin	−6.16	30.52	−7.15	5.75	−7.29	4.55	−7.16	5.66
	Orientin	−6.01	39.20	−6.53	16.31	−6.09	34.17	−6.00	39.70
	Isoorientin	−5.55	85.34	−6.92	8.50	−7.15	5.70	−6.57	15.30
	Hispidulin	−6.02	38.48	−7.63	2.53	−7.19	5.34	−5.99	40.61
	Tricin	−5.68	69.19	−7.82	1.84	−7.41	3.67	−5.94	44.46
Isoflavones	Genistein	−6.38	21.13	−7.27	4.72	−6.99	7.50	−6.40	20.48
	Daidzein	−6.64	13.47	−7.36	4.05	−6.99	7.52	−7.60	2.66
Flavonols	Quercetin	−5.51	91.56	−7.53	3.01	−7.08	6.41	−5.55	85.60
	Kaempferol	−5.99	40.73	−7.66	2.44	−7.65	2.49	−5.77	58.58
	Fisetin	−6.45	18.82	−8.77	0.37	−8.70	0.42	−5.85	51.23
	Myricetin	−5.03	205.46	−6.75	11.26	−6.60	14.60	−5.05	200.07
NOX inhibitor (setanaxib)		−8.35	0.76	na	na	na	na	na	na
XO inhibitor (febuxostat)		na	na	−6.94	8.18	−5.89	48.45	na	na
iNOS inhibitor(iNOS inhibitor-10)		na	na	na	na	na	na	−11.04	0.008

**Table 2:** Binding energies and inhibition constants (Ki) of apigenin and related compounds with IKK, p38 MAPK, NF-kB, and COX-2 obtains from AutoDock 4.2.6.

Sub- Family	Flavonoids	IKK PDB: 4KIK		p38 MAPK PDB: 4DLI		NF-kB (p50–p65) PDB: 1VKX		NF-kB (p52–p65) PDB: 3DO7		COX-2 PDB: 3NT1	
		Binding Energy (kcal/mol)	Ki (µM)	Binding Energy (kcal/mol)	Ki (µM)	Binding Energy (kcal/mol)	Ki (µM)	Binding Energy (kcal/mol)	Ki (µM)	Binding Energy (kcal/mol)	Ki (µM)
Flavones	Apigenin	−7.45	3.47	−8.21	0.96	−5.54	87.04	−6.16	30.54	−6.68	12.72
	Luteolin	−6.75	11.19	−8.21	0.95	−5.61	77.81	−6.34	22.43	−6.79	10.48
	Chrysin	−6.83	9.89	−8.40	0.70	−5.50	92.88	−6.33	22.84	−6.84	9.66
	Baicalein	−6.96	7.92	−8.31	0.80	−5.47	98.11	−6.15	31.29	−6.69	12.57
	Baicalin	−7.98	1.42	−9.91	0.05	−4.72	344.90	−5.75	61.09	−5.49	94.28
	Vitexin	−7.63	2.55	−9.29	0.15	−6.15	31.19	−5.72	63.95	−7.00	7.40
	Isovitexin	−7.85	1.77	−7.41	3.71	−5.14	172.13	−5.64	72.90	−6.34	22.52
	Orientin	−7.51	3.13	−9.25	0.17	−5.61	77.81	−5.83	53.14	−6.73	11.65
	Isoorientin	−7.70	2.27	−6.85	9.45	−6.10	33.82	−5.20	158.18	−5.40	109.50
	Hispidulin	−7.26	4.74	−7.99	1.38	−5.40	109.57	−6.21	27.93	−6.04	37.66
	Tricin	−6.84	9.65	−8.44	0.65	−5.24	143.80	−5.41	109.01	−6.70	12.27
Isoflavones	Genistein	−6.94	8.21	−8.08	1.19	−6.05	36.78	−5.89	48.27	−5.90	47.13
	Daidzein	−7.60	2.66	−7.76	2.03	−5.51	91.38	−5.96	42.65	−6.56	15.64
Flavonols	Quercetin	−6.74	11.43	−8.43	0.67	−5.04	203.17	−5.63	74.70	−6.09	34.53
	Kaempferol	−7.04	6.88	−8.19	0.99	−5.29	131.97	−5.95	43.51	−5.76	60.14
	Fisetin	−6.71	12.10	−8.14	1.08	−5.24	143.20	−5.94	44.59	−6.72	11.84
	Myricetin	−6.44	18.88	−8.34	0.77	−4.72	344.66	−5.30	130.94	−5.29	132.73
MAPK p38 inhibitor (adezmapimod)		na	na	−9.96	0.05	na	na	na	na	na	na
NF-kB inhibitor (sulfasalazine)		na	na	na	na	−6.99	7.49	−7.42	3.62	na	na
COX-2 inhibitor (naproxen)		na	na	na	na	na	na	na	na	−7.61	2.62

**Table 3:** Binding energies and inhibition constants (Ki) of apigenin and related compounds with EGFR and KRAS obtained from AutoDock 4.2.6.

Sub- Family	Flavonoids	EGFR (T790M/V948R) PDB: 6Z4B		KRAS G12C PDB: 4LV6 Switch I		KRAS G12C PDB: 4LV6 Switch II		KRAS G12D PDB: 6GJ8 Switch I		KRAS G12D PDB: 6GJ8 Switch II		KRAS G12V PDB:8AZZ Switch I		KRAS G12V PDB: 8AZZ Switch II	
		Binding Energy (kcal/mol)	Ki (µM)	Binding Energy (kcal/mol)	Ki (µM)	Binding Energy (kcal/mol)	Ki (µM)	Binding Energy (kcal/mol)	Ki (µM)	Binding Energy (kcal/mol)	Ki (µM)	Binding Energy (kcal/mol)	Ki (µM)	Binding Energy (kcal/mol)	Ki (µM)
Flavones	Apigenin	−7.41	3.67	−7.68	2.35	−6.47	17.97	−7.86	1.72	−5.68	69.12	−7.22	5.14	−6.57	15.24
	Luteolin	−7.64	2.50	−7.69	2.31	−6.37	21.47	−7.47	3.37	−5.25	142.33	−6.81	10.18	−6.65	13.36
	Chrysin	−7.35	4.07	−7.75	2.09	−6.56	15.58	−7.55	2.90	−5.74	61.73	−7.44	3.52	−7.19	5.41
	Baicalein	−6.76	11.08	−7.07	6.52	−6.67	12.81	−7.94	1.50	−5.27	136.05	−7.34	4.15	−7.37	3.98
	Baicalin	−8.08	1.19	−7.85	1.76	−8.28	0.849	−6.95	8.01	−5.37	115.45	−5.83	53.33	−7.72	2.21
	Vitexin	−8.04	1.27	−7.78	1.97	−6.76	11.08	−6.27	25.42	−5.72	64.64	−5.83	53.71	−6.65	13.29
	Isovitexin	−7.79	1.94	−7.14	5.85	−7.77	2.01	−7.89	1.64	−5.62	76.58	−6.21	28.03	−7.49	3.22
	Orientin	−7.88	1.68	−7.87	1.70	−7.18	5.42	−6.75	11.29	−4.66	381.43	−5.79	56.79	−6.21	28.00
	Isoorientin	−7.68	2.33	−6.83	9.89	−6.85	9.48	−6.80	10.44	−5.08	188.42	−5.93	45.13	−7.19	5.37
	Hispidulin	−7.03	7.08	−6.25	26.34	−6.03	38.27	−7.76	2.06	−5.00	215.17	−6.31	23.52	−6.63	13.87
	Tricin	−6.32	23.29	−6.95	7.99	−6.73	11.70	−6.89	8.85	−4.56	453.29	−5.93	45.15	−5.90	47.32
Isoflavone	Genistein	−6.55	15.76	−6.94	8.13	−6.53	16.33	−8.83	0.34	−5.31	127.81	−6.84	9.68	−7.95	1.50
	Daidzein	−7.06	6.63	−6.90	8.73	−6.97	7.73	−8.66	0.45	−5.20	154.75	−6.93	7.69	−7.26	4.76
Flavonols	Quercetin	−7.03	6.99	−7.09	6.39	−6.36	21.93	−7.62	2.59	−5.01	213.68	−6.17	29.81	−6.77	10.92
	Kaempferol	−7.28	4.62	−7.09	6.32	−6.79	10.50	−7.71	2.24	−5.13	174.36	−6.26	25.99	−6.82	10.08
	Fisetin	−6.45	18.68	−6.72	11.86	−6.48	17.91	−7.85	1.76	−5.40	110.47	−6.79	10.62	−7.36	4.02
	Myricetin	−6.61	14.21	−6.60	14.42	−6.12	32.43	−7.94	1.52	−4.49	514.89	−6.07	35.71	−6.55	15.87
EGFR inhibitor (erlotinib)		−8.86	0.32	na	na	na	na	na	na	na	na	na	na	na	na
EGFR inhibitor (gefitinib)		−8.20	0.97	na	na	na	na	na	na	na	na	na	na	na	na
KRAS inhibitor (adagrasib)		na	na	−11.31	0.005	−11.67	0.003	na	na	na	na	na	na	na	na
KRAS inhibitor (sotorasib)		na	na	−9.40	0.128	−8.95	0.28	na	na	na	na	na	na	na	na
KRAS inhibitor (MRTX1133)		na	na	na	na	na	na	−7.67	2.39	−7.49	3.22	na	na	na	na
KRAS inhibitor (BI-2865)		na	na	na	na	na	na	na	na	na	na	−6.97	7.74	−11.12	0.007

**Table 4:** Binding energies and inhibition constants (Ki) of apigenin and related compounds with BRAF, MEK-1, and ERK-1 obtains from AutoDock 4.2.6.

Sub- Family	Flavonoids	BRAF V599E PDB: 1UWJ		BRAF V600E PDB: 3OG7		MEK-1 C121S PDB: 7F2X		ERK-2 G169D PDB: 6D5Y	
		Binding Energy (kcal/mol)	Ki (µM)	Binding Energy (kcal/mol)	Ki (µM)	Binding Energy (kcal/mol)	Ki (µM)	Binding Energy (kcal/mol)	Ki (µM)
Flavones	Apigenin	−7.72	2.20	−6.93	8.36	−6.58	15.00	−5.66	71.20
	Luteolin	−6.99	7.50	−7.07	6.52	−6.23	27.28	−5.69	67.27
	Chrysin	−7.76	2.05	−6.89	8.93	−6.46	18.34	−5.31	129.07
	Baicalein	−7.54	2.99	−7.08	6.44	−6.47	18.11	−5.49	94.13
	Baicalin	−9.23	0.17	−7.72	2.21	−6.97	7.81	−6.41	20.08
	Vitexin	−8.09	1.17	−7.25	4.82	−6.11	33.08	−6.27	25.48
	Isovitexin	−8.10	1.16	−7.54	2.97	−6.85	9.54	−5.34	122.47
	Orientin	−7.31	4.35	−6.83	9.81	−6.12	32.74	−6.32	23.33
	Isoorientin	−7.85	1.75	−5.97	42.07	−6.91	8.65	−5.25	141.59
	Hispidulin	−6.64	13.53	−6.48	17.69	−6.25	26.08	−5.44	103.72
	Tricin	−6.88	9.05	−6.03	37.97	−7.07	6.56	−5.38	114.13
Isoflavone	Genistein	−6.96	7.93	−6.51	16.97	−6.78	10.73	−5.24	144.57
	Daidzein	−6.90	8.70	−6.93	8.34	−6.86	9.32	−5.43	105.32
Flavonols	Quercetin	−6.43	19.31	−5.59	79.26	−6.14	31.51	−5.49	95.16
	Kaempferol	−6.79	10.48	−5.99	40.34	−6.12	32.40	−5.33	123.20
	Fisetin	−7.06	6.73	−5.77	58.80	−6.42	19.80	−5.32	126.37
	Myricetin	−6.82	10.00	−5.05	197.92	−5.74	61.89	−5.14	171.93
RAF inhibitor (dabrafenib)		−13.27	1.87 × 10^−4^	−11.76	0.002	na	na	na	na
RAF inhibitor (encorafenib)		−10.57	0.02	−7.85	1.77	na	na	na	na
MEK inhibitor (trametinib)		na	na	na	na	−8.96	0.270	na	na
MEK inhibitor (selumetinib)		na	na	na	na	−6.81	10.23	na	na
ERK inhibitor (ulixertinib)		na	na	na	na	na	na	−7.85	1.75

**Table 5:** Binding energies and inhibition constants (Ki) of apigenin and related compounds with PI3K, AKT, and mTOR obtained from AutoDock 4.2.6.

Sub- Family	Flavonoids	PI3K PDB: 1E7U		AKT PDB: 3O96		mTOR PDB: 4JT6	
		Binding Energy (kcal/mol)	Ki (µM)	Binding Energy (kcal/mol)	Ki (µM)	Binding Energy (kcal/mol)	Ki (µM)
Flavones	Apigenin	−7.05	6.81	−6.57	15.26	−7.51	3.12
	Luteolin	−7.27	4.72	−6.71	12.12	−7.80	1.93
	Chrysin	−6.69	12.58	−6.80	10.40	−7.74	2.12
	Baicalein	−7.20	5.25	−6.42	19.64	−7.87	1.69
	Baicalin	−7.32	4.33	−8.27	0.86	−8.61	0.49
	Vitexin	−6.70	12.17	−8.64	0.46	−7.28	4.63
	Isovitexin	−5.40	110.84	−8.17	1.03	−7.07	6.53
	Orientin	−7.11	6.13	−8.19	0.99	−7.26	4.79
	Isoorientin	−5.39	112.16	−8.02	1.31	−7.37	3.97
	Hispidulin	−7.14	5.84	−6.49	17.57	−7.91	1.58
	Tricin	−7.37	3.96	−6.32	23.26	−7.40	3.79
Isoflavone	Genistein	−6.68	12.70	−6.46	18.53	−8.18	1.01
	Daidzein	−7.02	7.13	−6.67	12.84	−7.52	3.05
Flavonols	Quercetin	−6.93	8.31	−6.12	32.39	−7.07	6.62
	Kaempferol	−6.80	10.42	−6.27	25.57	−6.96	7.90
	Fisetin	−6.83	9.88	−6.73	11.58	−7.86	1.73
	Myricetin	−6.34	22.69	−6.03	38.21	−6.88	9.09
PI3K inhibitor (buparlisib)		−7.16	5.64	na	na	na	na
PI3K inhibitor (copanlisib)		−8.86	0.32	na	na	na	na
AKT inhibitor (GSK690693)		na	na	−9.73	0.07	na	na
mTOR inhibitor (PI-103)		na	na	na	na	−9.22	0.15

**Table 6:** Binding energies and inhibition constants (Ki) of apigenin and related compounds with CDK2, CDK4, and CDK6 obtained from AutoDock 4.2.6.

Sub- Family	Flavonoids	CDK2 PDB: 1GIH		CDK2 PDB: 4ERW		CDK2 PDB: 1AQ1		CDK4 PDB: 3G33		CDK4 PDB: 2W96		CDK6 PDB:1XO2		CDK6 PDB: 5L2T	
		Binding Energy (kcal/mol)	Ki (µM)	Binding Energy (kcal/mol)	Ki (µM)	Binding Energy (kcal/mol)	Ki (µM)	Binding Energy (kcal/mol)	Ki (µM)	Binding Energy (kcal/mol)	Ki (µM)	Binding Energy (kcal/mol)	Ki (µM)	Binding Energy (kcal/mol)	Ki (µM)
Flavones	Apigenin	−7.21	5.17	−6.62	14.07	−6.88	9.10	−5.34	121.78	−6.64	13.66	−7.80	1.75	−8.73	0.40
	Luteolin	−7.18	5.48	−6.42	19.74	−6.85	9.53	−5.75	60.80	−6.53	16.42	−7.92	1.57	−8.14	1.07
	Chrysin	−7.23	5.02	−6.91	8.60	−7.16	5.69	−5.21	151.49	−6.67	12.89	−7.75	2.10	−8.37	0.73
	Baicalein	−7.03	6.98	−6.55	15.79	−6.99	7.51	−5.75	60.86	−6.50	17.07	−8.10	1.15	−8.40	0.70
	Baicalin	−7.33	4.21	−8.69	0.43	−8.32	0.79	−6.41	20.17	−6.74	11.49	−8.46	0.63	−8.60	0.50
	Vitexin	−8.78	0.37	−7.67	2.40	−6.91	8.58	−6.45	18.78	−6.75	11.27	−9.77	0.07	−7.14	5.86
	Isovitexin	−8.20	0.97	−8.26	0.88	−7.18	5.50	−4.82	294.02	−6.33	23.03	−8.95	0.27	−9.77	0.07
	Orientin	−8.46	0.63	−7.70	2.25	−7.25	4.88	−6.21	27.89	−6.49	17.57	−9.35	0.14	−6.92	8.41
	Isoorientin	−7.79	1.95	−7.92	1.51	−7.38	3.89	−5.14	170.04	−5.89	47.98	−8.97	0.27	−8.81	0.35
	Hispidulin	−6.97	7.80	−6.78	10.78	−6.89	8.85	−5.54	87.47	−5.97	41.76	−8.05	1.25	−8.39	0.71
	Tricin	−7.48	3.28	−6.92	8.40	−6.61	14.24	−5.24	144.80	−7.04	6.90	−7.62	2.61	−7.27	4.72
Isoflavones	Genistein	−6.70	12.28	−6.75	11.20	−6.24	26.83	−5.50	92.47	−6.45	18.72	−7.67	2.40	−8.21	0.96
	Daidzein	−7.38	3.88	−6.54	16.00	−6.91	8.61	−5.65	68.52	−6.61	14.21	−7.64	2.53	−8.40	0.70
Flavonols	Quercetin	−6.58	15.03	−6.35	22.27	−6.64	13.56	−5.82	54.35	−5.89	48.55	−8.13	1.11	−7.49	3.21
	Kaempferol	−7.00	7.40	−6.79	10.52	−6.40	20.41	−5.38	113.87	−6.41	20.11	−7.92	1.57	−8.03	1.29
	Fisetin	−7.03	7.05	−6.64	13.50	−6.99	7.50	−5.90	47.35	−6.50	17.33	−8.55	0.54	−6.99	7.50
	Myricetin	−6.20	28.44	−6.14	31.34	−6.29	24.69	−5.54	86.32	−5.94	44.44	−8.58	0.51	−6.94	8.21
CDK 2 inhibitor (1PU)		−9.18	0.19	−8.68	0.44	−8.47	0.62	na	na	na	na	na	na	na	na
CDK 4 inhibitor (palbociclib)		na	na	na	na	na	na	−8.44	0.65	−9.51	0.11	na	na	na	na
CDK 6 inhibitor (ribociclib)		na	na	na	na	na	na	na	na	na	na	−10.32	0.03	−10.29	0.03

**Table 7:** Binding energies and inhibition constants (Ki) of apigenin and related compounds with Aromatase, DMNT1, HDAC1, and HDAC2 obtained from AutoDock 4.2.6.

Sub- Family	Flavonoids	Aromatase PDB: 3EQM		Aromatase PDB: 5JKV		DNMT1 PDB: 3AV6		HDAC1 PDB: 4BKX		HDAC2 PDB: 4LXZ	
		Binding Energy (kcal/mol)	Ki (µM)	Binding Energy (kcal/mol)	Ki (µM)	Binding Energy (kcal/mol)	Ki (µM)	Binding Energy (kcal/mol)	Ki (µM)	Binding Energy (kcal/mol)	Ki (µM)
Flavones	Apigenin	−6.51	16.90	−6.50	17.33	−7.80	1.92	−6.82	10.08	−7.10	6.25
	Luteolin	−6.37	21.47	−6.19	28.89	−7.66	2.41	−6.85	9.50	−6.42	19.63
	Chrysin	−6.45	18.71	−6.66	13.04	−6.92	8.44	−7.25	4.81	−6.93	8.37
	Baicalein	−6.83	9.84	−6.36	21.96	−7.01	7.27	−6.68	12.61	−5.95	43.19
	Baicalin	−7.53	3.01	−6.58	14.69	−8.49	0.60	−6.34	22.59	−6.10	33.65
	Vitexin	−7.15	5.77	−6.21	28.19	−8.03	1.30	−5.91	46.78	−5.88	48.72
	Isovitexin	−6.33	23.05	−6.85	9.60	−6.97	7.82	−4.36	634.05	−3.93	1320
	Orientin	−5.82	53.85	−6.61	14.20	−6.98	7.68	−5.81	55.45	−4.96	232.05
	Isoorientin	−5.62	75.78	−5.03	206.28	−6.26	25.60	−4.45	551.34	−4.03	1120
	Hispidulin	−5.94	44.44	−5.68	68.38	−7.77	2.02	−6.19	29.14	−5.74	61.90
	Tricin	−5.82	54.07	−5.82	53.91	−7.58	2.77	−6.48	17.65	−5.89	48.24
Isoflavones	Genistein	−6.51	16.96	−6.50	17.10	−7.34	4.17	−5.50	93.27	−5.36	118.61
	Daidzein	−6.70	12.32	−6.63	13.87	−7.06	6.74	−6.29	24.48	−6.59	14.73
Flavonols	Quercetin	−5.79	57.03	−5.62	75.60	−7.54	2.99	−4.73	338.65	−4.98	222.77
	Kaempferol	−5.87	50.02	−5.87	49.55	−7.57	2.84	−5.45	100.60	−5.19	156.74
	Fisetin	−6.29	24.47	−6.29	24.56	−8.12	1.12	−5.49	94.40	−5.13	174.33
	Myricetin	−5.31	128.69	−5.02	207.32	−7.41	3.69	−4.38	617.88	−5.21	152.93
Aromatase inhibitor (letrozole)		−7.77	2.03	−7.77	2.01	na	na	na	na	na	na
DNMT 1 inhibitor (azacytidine)		na	na	na	na	−7.41	3.73	na	na	na	na
HDAC 1&2 inhibitor (vorinostat)		na	na	na	na	na	na	−7.49	3.22	−9.19	0.18

**Table 8:** Binding energies and inhibition constants (Ki) of apigenin and related compounds with DNA gyrase B and FABZ obtained from AutoDock 4.2.6.

Sub- Family	Flavonoids	DNA gyrase B PDB: 6F86		DNA gyrase B PDB: 4PRV		FABZ PDB: 3CF9 Site A		FABZ PDB: 3CF9 Site B	
		Binding Energy (kcal/mol)	Ki (µM)	Binding Energy (kcal/mol)	Ki (µM)	Binding Energy (kcal/mol)	Ki (µM)	Binding Energy (kcal/mol)	Ki (µM)
Flavones	Apigenin	−6.23	27.21	−6.51	16.97	−7.70	2.27	−7.98	1.42
	Luteolin	−5.74	61.92	−6.39	20.80	−8.19	0.98	−7.74	2.11
	Chrysin	−6.66	13.03	−6.83	9.8	−7.75	2.09	−7.78	1.99
	Baicalein	−6.24	26.84	−6.43	19.45	−7.85	1.77	−7.45	3.45
	Baicalin	−6.24	26.48	−8.61	0.49	−6.90	8.79	−9.81	0.06
	Vitexin	−5.37	115.48	−7.34	4.18	−7.52	3.07	−9.44	0.12
	Isovitexin	−6.00	39.75	−7.29	4.51	−7.32	4.30	−8.36	0.74
	Orientin	−5.13	173.59	−7.13	5.94	−6.99	7.50	−8.96	0.27
	Isoorientin	−5.25	142.45	−7.75	2.07	−7.05	6.84	−8.89	0.30
	Hispidulin	−6.10	33.55	−6.52	16.65	−7.72	2.20	−7.48	3.27
	Tricin	−6.81	10.14	−6.22	27.59	−7.13	5.96	−8.06	1.23
Isoflavones	Genistein	−5.46	99.68	−6.75	11.33	−8.32	0.80	−8.74	0.39
	Daidzein	−5.72	64.48	−7.31	4.40	−7.75	2.10	−8.21	0.96
Flavonols	Quercetin	−5.31	128.9	−6.64	13.69	−7.36	4.01	−7.29	4.57
	Kaempferol	−5.29	131.63	−6.26	25.60	−7.52	3.07	−7.03	6.98
	Fisetin	−5.83	53.49	−7.26	4.77	−7.53	3.05	−7.55	2.90
	Myricetin	−5.66	70.99	−6.07	35.77	−7.08	6.51	−6.98	7.65
Antibiotic (ciprofloxacin)		−7.88	1.66	−10.45	0.02	−9.97	0.05	−11.68	0.002

## Data Availability

Data are provided within the manuscript or Supplementary Information Files.

## Supplementary Materials

The supporting information can be downloaded at: https://www.mdpi.com/article/10.3390/ijms26062548/s1.
