# Supplementary material for: Evaluation of the Inhibitory Potential of Apigenin and Related Flavonoids on Various Proteins Associated with Human Diseases Using AutoDock
Source: Int J Mol Sci. 2025 Mar 12;26(6):2548. doi: 10.3390/ijms26062548 (PMC11942390; doi:10.3390/ijms26062548)

Figure S1 Genetic Algorithm Parameters used in molecular docking

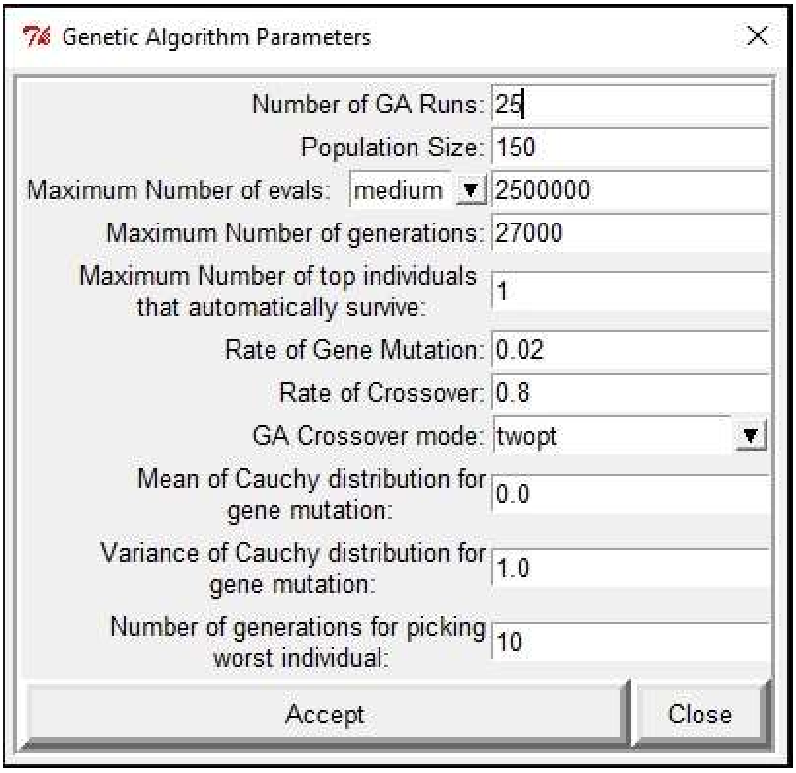

Supplement: Supplementary file 1 [file ijms-26-02548-s001.zip › Figure S1 Genetic Algorithm Parameters using in molecular docking.pdf]
