# Supplementary material for: Evaluation of the Inhibitory Potential of Apigenin and Related Flavonoids on Various Proteins Associated with Human Diseases Using AutoDock
Source: Int J Mol Sci. 2025 Mar 12;26(6):2548. doi: 10.3390/ijms26062548 (PMC11942390; doi:10.3390/ijms26062548)
Supplement: Supplementary file 1 [file ijms-26-02548-s001.zip › Table S1 List of the ligands (flavonoids) selected for this study.pdf]

Table S1 List of the ligands (flavonoids) selected for this study

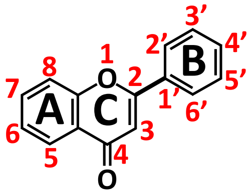

Flavone

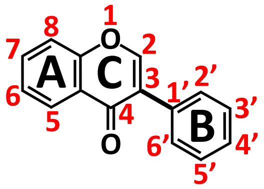

Isoflavone

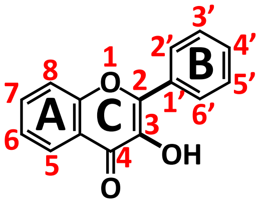

Flavonol

Subclass of flavonoid	Name (compound ID)	Chemical structure	Functional group										
			A ring				B ring					C ring	
			5	6	7	8	2'	3'	4'	5'	6'	3'	Double bond
**Flavone**	Apigenin (5280443)	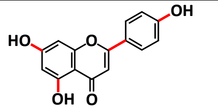	-OH		-OH				-OH				1
	Luteolin (5280445)	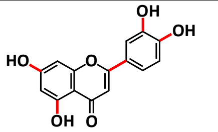	-OH		-OH			-OH	-OH				1
	Chrysin (5281607)	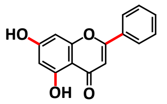	-OH		-OH								1
	Baicalein (5281605)	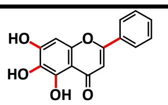	-OH	-OH	-OH								1
	Baicalin (64982)	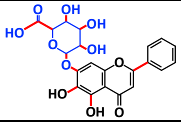	-O- GLU	-OH	-OH								1
	Vitexin (5280441)	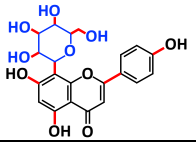	-OH		-OH	-C- GLU			-OH				1
	Isovitexin (162350)	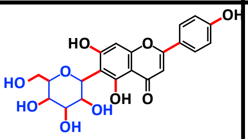	-OH	-C- GLU	-OH				-OH				1
	Orientin (5281675)	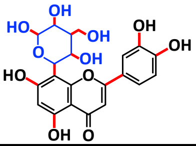	-OH		-OH	-C- GLU		-OH	-OH				1

	Isoorientin (114776)	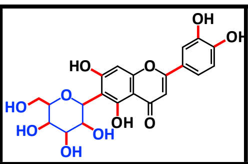	-OH	-O-GLU	-OH			-OH	-OH				1
	Hispidulin (5281628)	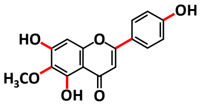	-OH	-O-CH3	-OH				-OH				1
	Tricin (5281702)	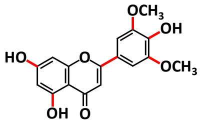	-OH		-OH			-O-CH3	-OH	-O-CH3			1
**Isoflavone**	Genistein (5280961)	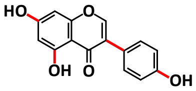	-OH		-OH				-OH				1
	Daidzein (5281708)	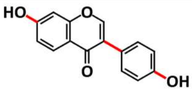			-OH				-OH				1
**Flavonol**	Quercetin (5280343)	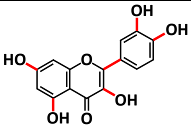	-OH		-OH			-OH	-OH			-OH	1
	Kaempferol (5280863)	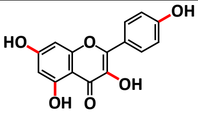	-OH		-OH				-OH			-OH	1
	Fisetin (5281614)	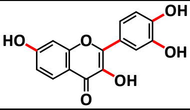			-OH				-OH	-OH		-OH	1
	Myricetin (5281672)	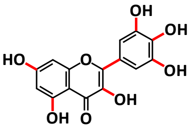	-OH		-OH			-OH	-OH	-OH		-OH	1
