# Supplementary material for: Evaluation of the Inhibitory Potential of Apigenin and Related Flavonoids on Various Proteins Associated with Human Diseases Using AutoDock
Source: Int J Mol Sci. 2025 Mar 12;26(6):2548. doi: 10.3390/ijms26062548 (PMC11942390; doi:10.3390/ijms26062548)
Supplement: Supplementary file 1 [file ijms-26-02548-s001.zip › Table S2 Description of receptors (proteins).pdf]

Table S2 Description of receptors (proteins)

	Name	Description
**Oxidative stress**		
1	Nicotinamide adenine dinucleotide phosphate (NADPH) oxidase (NOX)	Production of superoxide anion and reactive oxygen species
2	Xanthine oxidase (XO)	Production of reactive oxygen species
3	inducible nitric oxide synthase (iNOS)	Reactive oxygen and nitrogen metabolite-metabolizing enzyme
**Inflammation**		
1	Inhibitor of nuclear factor-kappa B (IκB) kinase (IKK)	A serine/threonine protein kinase that phosphorylates the I-kappa-B protein to activate NF-κB
2	p38 Mitogen-activated protein kinases (p38 MAPK)	A class of mitogen-activated protein kinases.<br>Involvement in inflammation
3	Nuclear factor-kappa B (NF-κB)	Transcription factor, a key regulator in the production of cytokines and chemokines
4	Cyclooxygenase enzyme-2 (COX-2)	Association with pro-inflammatory stimuli, such as cytokines, growth factors, and tumor promoters
**Carcinogenesis**		
1	Epidermal growth factor receptor (EGFR)	Regulation of epithelial cell proliferation, survival, differentiation, migration, inflammatory processes, and extracellular matrix<br>The driver of carcinogenesis (inappropriate activation)
2	Kirsten rat sarcoma viral oncoprotein homolog (KRAS)	Regulation of cell division and proliferation<br>Carcinogenesis.
3	Rapidly accelerated fibrosarcoma (RAF)	Transduction of mitogenic signals from the cell membrane to the nucleus<br>Serine/threonine kinases
4	Mitogen-activated protein kinase kinase 1 (MEK-1)	Regulation of cell cycle progression
5	Extracellular signal-regulated kinase 2 (ERK-2)	Involvement in tumor cell proliferation, differentiation, adhesion, migration, and survival.

6	Phosphatidylinositol 3-kinase (PI3K)	Cell growth, proliferation, and differentiation
7	A cellular homolog of murine thymoma virus akt8 oncogene (AKT)	Serine-threonine kinase
8	Mammalian target of rapamycin (mTOR)	The protein binds to rapamycin, Association with tumor cell proliferation helps control several cell functions, including cell division and survival
9	Cyclin-dependent kinase 2 (CDK2),	Serine/threonine protein kinase Cell cycle regulation
10	Cyclin-dependent kinase 4 (CDK4),	Serine/threonine protein kinase Cell cycle regulation
11	Cyclin-dependent kinase 6 (CDK6),	Serine/threonine protein kinase Cell cycle regulation
12	Aromatase	Conversion of androgen to estrogen
13	DNA methyltransferases-1 (DNMT1)	Transfer of a methyl group from the methyl donor, S-adenosyl-L-methionine (SAM), to the 5-position of cytosine residues in the DNA molecule
14	Histone-acetylase 1 (HDAC1)	Regulation of the cell cycle, angiogenesis, apoptosis, and differentiation
15	Histone-acetylase 2 (HDAC2)	Regulation of the cell cycle, angiogenesis, apoptosis, and differentiation
**Bacterial Infection**		
1	Bacterial DNA gyrase B	Uncoiling the supercoiled DNA helix
2	3R-hydroxy acyl-ACP dehydratase (FABZ)	Fatty acid synthesis
