# Supplementary material for: Evaluation of the Inhibitory Potential of Apigenin and Related Flavonoids on Various Proteins Associated with Human Diseases Using AutoDock
Source: Int J Mol Sci. 2025 Mar 12;26(6):2548. doi: 10.3390/ijms26062548 (PMC11942390; doi:10.3390/ijms26062548)
Supplement: Supplementary file 1 [file ijms-26-02548-s001.zip › Table S3 Information of proteins and grid parameters.pdf]

Table S3 Information of proteins and grid parameters

Receptor	Source PBD_ID	Grid parameter		Animo acid residue (using for localization of pocket)	References
		Grid center	Grid size		
**Oxidative stress**					
NOX	2CDU	x = -0.152<br>y = 9.968<br>z = 52.315	55 x 55 x 55	TYR 159, ILE 160, ASP 179, HIS 181, TYR 188, LYS 213, VAL 214, ILE 243	PMID: 16893166
XO	1FIQ	x = -28.571<br>y = 10.401<br>z = 115.552	60 x 60 x 60	LEU 648, PHE 649, LEU 873, ARG 880, PHE 914, PHE 1009, THR 1010, VAL 1011, PHE 1013, LEU 1014, PRO 1076, GLU 1261	PMID: 12054758
XO	3ETR	x = 8.654<br>y = -23.952<br>z = 25.541	50 x 50 x 50	LEU 648, PHE 649, ASN 768, ASN 768, LYS 771, GLU 802, LEU 873, HIS 875, SER 876, ARG 880, PHE 914, PHE 1009, THR 1010, VAL 1011, PHE 1013, LEU 1014	PMID: 26285120
iNOS	4NOS	x = 31.408<br>y = 98.740<br>z = 16.743	55 x 55 x 55	ARG 199, TYR 347, TYR 373, ASP 382, ARG 388, TRP 463	DOI: 10.26434/chemrxiv-2022-276cm
**Inflammation**					
IKK	4KIK	x = -13.516<br>y = -31.516<br>z = -74.982	55 x 55 x 55	Leu 21, ALA 42, CYS 99, GLU 149, ILE 165	DOI: 10.26434/chemrxiv-2022-276cm
p38 MAPK	4DLI	x = 23.292<br>y = -14.889<br>z = -9.325	50 x 50 x 50	GLU 192, ASN 196, SER 251, ASP 292, ASP 294	https://www.omicsonline.org/open-access-pdfs/molecular-docking-studies-on-apigenin-as-a-target-with-mapk-p38-forcardiovascular-diseases-.pdf
NF-kB (p50-p65)	1VKX (DNA binding site)	x = -1.779<br>y = 28.024<br>z = 60.659	95 x 95 x 90	p50: ARG 354, ARG 356, TYR 357, CYS 359, GLU 260, GLN 574, ARG 605<br>p65:<br>ARG 33, ARG 35, TYR 36, CYS 38, GLU 39, LYS 122, LYS 123, ARG 187, LYS 218, GLN 220, LYS 221, ARG 246, GLN 247	PMID: 25964540
NF-kB (p52-p65)	3DO7 (DNA binding site)	x = 27.108<br>y = 60.348<br>z = 75.486	90 x 94 x 100	p52: Chain B<br>ARG 52, ARG 54, TYR 55, CYS 57, GLU 58, SER 61, HIS 62, THR 142, LYS 143, LYS 252, GLN 254, LYS 255, GLN 284<br>RelB: Chain A<br>ARG 117, ARG 119, TYR 120, CYS 122, GLU 123, ARG 125, SER 129, ARG 209, LYS 210,	PMID: 25964540

Receptor	Source PBD_ID	Grid parameter		Animo acid residue (using for localization of pocket)	References
		Grid center	Grid size		
				GLN 307, LYS 308, ARG 333, GLN 334	
COX-2	3NT1	x = -36.935<br>y = -50.935<br>z = -22.160	60 x 60 x 60	ARG 120, VAL 349, LEU 352, TYR 355, LEU 384, TYR 385, TRP 387, MET 522, VAL 523, ALA 527, SER 530, LEU 531	PMID: 34345856
**Carcinogenesis**					
EGFR	6Z4B	x = 42.339<br>y = -9.112<br>z = -5.245	55 x 55 x 55	LEU 718, ALA 743, CYS 797, GLN 791, LEU 792, MET 793, LEU 844, THR 854	DOI: 10.1039/D2NJ00389A
KRAS (G12C) Switch I	4LV6	x = -4.461<br>y = -14.847<br>z = 15.336	64 x 70 x 85	CYS 12, GLN 25, ASN 26, HIS 27, PHE 28, VAL 29, ASP 30, GLU 31, TYR 32, ASP 33, PRO 34, THR 35, ILE 36, GLU 37, ASP 38, SER 39, TYR 40	PMID: 24256730<br><br>DOI: 10.3390/cimb45030137
KRAS (G12C) Switch II	4LV6	x = -8.522<br>y = -2.400<br>z = 8.523	58 x 71 x 73	CYS 12, ASP 57, THR 58, ALA 59, GLY 60, GLN 61, GLU 62, GLU 63, TYR 64, SER 65, ALA 66, MET 67, ARG 68, ASP 69, GLN 70, TYR 71, MET 72, ARG 73, THR 74, GLY 75, GLU 76	PMID: 24256730<br><br>DOI: 10.3390/cimb45030137
KRAS (G12D) Switch I	6GJ8	x = 12.77<br>y = 30.167<br>z = -14.483	60 x 60 x 60	ASP 12, GLN 25, ASN 26, HIS 27, PHE 28, VAL 29, ASP 30, GLU 31, TYR 32, ASP 33, PRO 34, THR 35, ILE 36, GLU 37, ASP 38, SER 39, TYR 40	PMID: 31332011<br><br>DOI: 10.3390/cimb45030137
KRAS (G12D) Switch II	6GJ8	x = 7.613<br>y = 11.81<br>z = -18.035	60 x 60 x 60	ASP 12, ASP 57, THR 58, ALA 59, GLY 60, GLN 61, GLU 62, GLU 63, TYR 64, SER 65, ALA 66, MET 67, ARG 68, ASP 69, GLN 70, TYR 71, MET 72, ARG 73, THR 74, GLY 75, GLU 76	PMID: 31332011<br><br>DOI: 10.3390/cimb45030137
KRAS (G12V) Switch I	8AZZ	x = 17.455<br>y = -2.491<br>z = 23.755	70 x 70 x70	VAL 12, GLN 25, ASN 26, HIS 27, PHE 28, VAL 29, ASP 30, GLU 31, TYR 32, ASP 33, PRO 34, THR 35, ILE 36, GLU 37, ASP 38, SER 39, TYR 40	PMID: 37258666<br><br>DOI: 10.3390/cimb45030137
KRAS (G12V) Switch II	8AZZ	x = 14.103<br>y = -2.491<br>z = 7.136	80 x 70 x 60	VAL 12, ASP 57, THR 58, ALA 59, GLY 60, GLN 61, GLU 62, GLU 63, TYR 64, SER 65, ALA 66, MET 67, ARG 68, ASP 69, GLN 70, TYR 71, MET 72, ARG 73, THR 74, GLY 75, GLU 76	PMID: 37258666<br><br>DOI: 10.3390/cimb45030137
BRAF (V599E)	1UWJ	x = 1.737<br>y = 23.035<br>z = 32.687	60 x 60 x 60	ALA 480, GLU 500, ILE 512, THR 528, GLN 529, TRP 530, CYS 531, LEU 566, HIS 573, ILE 591, ASP 593	PMID: 15035987

Receptor	Source PBD_ID	Grid parameter		Animo acid residue (using for localization of pocket)	References
		Grid center	Grid size		
BRAF (V600E)	3OG7	x = -2.041<br>y = 0.199<br>z = -25.096	60 x 60 x 60	ALA 481, LYS 483, LEU 505, LEU 514, THR 529, GLN 530, TRP 531, CYS 532, ASP 594, PHE 595	PMID: 20823850
MEK-1 (C121S)	7F2X	x = -10.039<br>y = 16.737<br>z = 29.297	65 x 80 x 55	ASN 78, VAL 82, ALA 95, LYS 97, MET 143, GLU 144, MET 146, SER 150, ARG 189, LYS 192, SER 194, LEU 197, GLY 210, VAL 211, SER 212, LEU 215, ILE 216, MET 219, ARG 234	PMID: 35831322
ERK-2 (G169D)	6D5Y	x = 18.113<br>y = 3.467<br>z = 19.411	75 x 60 x 60	TYR 30, ILE 31, GLY 34, ALA 35, TYR 36, VAL 39, SER 41, ARG 50, ALA 52, LYS 54, GLU 71, ILE 84, GLN 105, ASP 106, LEU 107, MET 108, GLU 109, THR 110, ASP 111, LYS 114, LYS 151, SER 153, ASN 154, LEU 156, CYS 166, ASP 167	DOI: 10.13005/bpj/2331<br><br>PMID: 29760222<br><br>PMID: 34395292
PI3K	1E7U	x = 23.692<br>y = 60.132<br>z = 21.571	50 x 50 x 50	MET 804, SER 806, PRO 810, LEU 811, TRP 812, ILE 831, PHE 832, LYS 833, ASP 836, LEU 838, TYR 867, ILE 879, ILE 881, VAL 882, ASN 951, MET 953, ILE 954, PHE 961, ILE 963	PMID: 11090628
AkT	3O96	x = 6.865<br>y = -5.287<br>z = 11.964	50 x 50 x 50	ASN 53, TRP 80, THR 81, THR 82, SER 205, LYS 268, TYR 272, ARG 273, ASP 292	DOI: 10.18311/jnr/2022/28194<br><br>PMID: 35340883
mTOR	4JT6	x = -17.590<br>y = -33.326<br>z = -55.676	55 x 55 x 55	LEU 2185, GLU 2190, ASP 2195, TYR 2225, VAL 2240, ASP 2357	DOI: 10.15406/mojpb.2017.05.00177
CDK2	1GIH	x = 8.778<br>y = 8.915<br>z = 24.05	50 x 50 x 50	ILE 10, VAL 18, ALA 31, LYS 33, VAL 64, PHE 80, PHE 82, LEU 83, HIS 84, GLN 85, ASP 86, LEU 134, ALA 144, ASP 145	PMID: 11335721<br><br>PMID: 25870953
CDK2	4ERW	x = -1.383<br>y = -6.333<br>z = 26.250	55 x 55 x 55	ILE 10, VAL 18, LYS 33, PHE 80, GLU 81, PHE 82, LEU 83, HIS 84, GLN 85, ASP 86, ASN 132, LEU 134, ASP 145	PMID: 25870953
CDK2	1AQ1	x = 0.101<br>y = 27.292<br>z = 9.246	60 x 60 x 60	LYS 9, ILE 10, GLY 11, GLU 12, THR 14, VAL 18, VAL 64, GLU 81, PHE 82, LEU 83, LYS 129, GLN 131, LEU 134, ALA 144, ASP 145	DOI: 10.14744/ejmo.2022.39682
CDK4	3G33	x = 1.210<br>y = -30.683<br>z = -53.989	95 x 75 x 90	CYS 68 (B), GLU 70 (B), GLN 71 (B), ARG 72 (B), CYS 73 (B), GLU 74 (B), GLU 75 (B), PHE 78 (B), LEU 108 (B), LYS 112 (B),	PMID: 34421361<br><br>PMID: 33477856

Receptor	Source PBD_ID	Grid parameter		Animo acid residue (using for localization of pocket)	References
		Grid center	Grid size		
				THR 120 (B), ILE 121 (B), HIS 158 (B), PRO 45 (C), GLY 48 (C), GLY 50 (C), GLY 51 (C), GLY 52 (C), GLY 53 (C), LEU 54 (C), PRO 55 (C), ILE 56 (C), SER 57 (C), THR 58 (C), ARG 168 (C), SER 171 (C), TYR 172 (C), CYS 68 (D), GLN 71 (D), ARG 72 (D),CYS 73 (D), GLU 74 (D), GLU 75 (D), PHE 78 (D), GLU 115 (D), THR 116 (D), THR 117 (D), LEU 119 (D), LYS 123 (D), HIS 158 (D)	PMID: 30917530
CDK4	2W96	x = 5.936<br>y = -0.097<br>z = 79.728	60 x 60 x 60	GLY 15, ALA 16, VAL 20, LYS 35, HIS 95, VAL 96, ASP 97, ASP 99, ARG 101, GLU 144, ASN 145, LEU 147, ASP 158	https://phcog.com/article/view/2020/16/69/258-263
CDK6	1XO2	x = 1.615<br>y = 37.439<br>z = 140.013	55 x 55 x 55	ILE 19, ALA 41, LYS 43, GLU 61, PHE 98, GLU 99, HIS 100, VAL 101, ASP 104, GLN 149, LEU 152, ASP 163	PMID: 15689157
CDK6	5L2T	x = 24.662<br>y = 42.061<br>z = -2.029	70 x 70 x 70	GLU 21, GLY 22, ALA 23, VAL 27, LYS 29, ALA 41, LYS 43, ARG 44, VAL 45, ARG 46, LYS 147, GLN 149, ASP 163, LEU 166, ALA 167	DOI: 10.14744/ejmo.2022.39682
Aromatase	3EQM	x = 86.127<br>y = 55.392<br>z = 43.623	55 x 55 x 55	ARG 115, ILE 133, PHE 134, TRP 224, LEU 228, ILE 305, ALA 306, ASP 309, THR 310, VAL 370, LEU 372, VAL 373, MET 374, ARG 435, LEU 477	PMCID: PMC6263336
Aromatase	5JKV	x = 85.678<br>y = 56.391<br>z = 47.843	55 x 55 x 55	ARG 115, ILE 133, PHE 134, PHE 211, TRP 224, ALA 306, ASP 309, THR 310, VAL 369, VAL 370, LEU 372, VAL 373, MET 374, SER 478	PMID: 37233624
DNMT1	3AV6	x = 75.034<br>y = 16.320<br>z = 39.169	80 x 80 x 80	LEU 1145, ASP 1146, VAL 1147, TRP 1168, ALA1169, ILE 1170, LEU 1223, CYS 1224, GLY 1225, GLY 1226, PRO 1227, PRO 1228, TYR 1243, SER 1244, PHE 1265, PHE 1266, LEU 1267, LEU 1268, ASN 1270, VAL 1271, THR 1312, VAL 1578, ASN 1580	PMID: 27658199
HDAC1	4BKX	x = -45.498<br>y = 17.411<br>z = -5.177	50 x 50 x 50	Chain B<br>MET 30, ASP 99, LEU 139, HIS 140, HIS 141, GLY 149, PHE 150, CYS 151, ASP 176, HIS 178, PHE 205, GLN 260, ASP 264,	PMID: 33291755

Receptor	Source PBD_ID	Grid parameter		Animo acid residue (using for localization of pocket)	References
		Grid center	Grid size		
				LEU 271, GLY 300, GLY 301, TYR 303	
HDAC2	4LXZ	x = 21.677<br>y = -18.008<br>z = 1.197	45 x 45 x 45	Chain A<br>MET 35, ASP 104, GLY 143, LEU 144, HIS 145, HIS 146, GLY 154, PHE 155, CYS 156, ASP 181, HIS 183, PHE 210, PRO 211, GLN 265, ASP 269, LEU 276, GLY 305, GLY 306, GLY 307, TYR 308	PMID: 33291755
**Infection**					
DNA gyrase B	6F86	x = 55.658<br>y = 22.889<br>z = 62.369	60 x 60 x 60	VAL 43, ASN 46, ALA 47, ASP 49, GLU 50, ASP 73, ARG 76, GYL 77, ILE 78, PRO 79, ILE 94, LEU 98, VAL 120, ARG 136, THR 165, VAL 167	PMID: 33980935
DNA gyrase B	4PRV	x = 14.534<br>y = 21.944<br>z = -9.615	55 x 55 x 55	ASN 46, GLU 50, ASP 73, ILE 78, ILE 94, LYS 103, TYR 109, LEU 115, HIS 116, VAL 118, VAL 120	PMID: 27049530<br>PMID: 25202966
FABZ Site A	3CF9	x = 18.644<br>y = 17.322<br>z = -11.883	45 x 45 x 45	HIS 58 (A), PHE 59 (A), LYS 62 (A), ILE 64 (A), ARG 110 (A), ILE 111 (A), PRO 112 (A), ILE 98 (B), TYR 100 (B), GLU 159 (B)	PMID: 18780820
FABZ Site B	3CF9	x = 4.611<br>y = -10.376<br>z = -35.701	45 x 45 x 45	ILE 20 (C), LEU 21 (C), PRO 22 (C), HIS 23 (C), GLN 76 (C), PHE 80 (C), PHE 83 (C), LYS 97 (C), ILE 98 (C), VAL 99 (C), TYR 100 (C), PHE 101 (C), ARG 158 (C), PHE 59 (D), LYS 62 (D), ILE 64 (D)	PMID: 18780820
