# Supplementary material for: Evaluation of the Inhibitory Potential of Apigenin and Related Flavonoids on Various Proteins Associated with Human Diseases Using AutoDock
Source: Int J Mol Sci. 2025 Mar 12;26(6):2548. doi: 10.3390/ijms26062548 (PMC11942390; doi:10.3390/ijms26062548)
Supplement: Supplementary file 1 [file ijms-26-02548-s001.zip › Table S4 Additional grid map for each drugs or inhibitors.pdf]

Table S4 Additional grid map created specific for each drugs or inhibitors

Drugs/inhibitors	Additional grid map
Setanaxib (NOX Inhibitor)	Cl
Febuxostat (XO Inhibitor)	S
iNOSos Inhibitor-10 (iNOS Inhibitor)	S
Adezmapimod (MAPK P38 Inhibitor)	F, S
Sulfasalazine (NF-Kb Inhibitor)	S
Naproxen (COX-2 Inhibitor)	-
Erlotinib (EGFR Inhibitor)	-
Getifinib EGFR Inhibitor)	Cl, F
Adagrasib (Kras Inhibitor)	Cl, F
Sotorasib (Kras Inhibitor)	F
MRTX1133 (Kras Inhibitor)	F
BI-2865 (Kras Inhibitor)	S
Dabrafenib (RAF Inhibitor)	F, S
Encorafenib (RAF Inhibitor)	Cl, F, S
Trametinib (MEK Inhibitor)	F, I
Selumetinib (MEK Inhibitor)	Br, Cl, F
Ulixertinib (ERK Inhibitor)	Cl
Buparlisib (PI3K Inhibitor)	F
Copanlisib (PI3K Inhibitor)	-
GSK690693 (AKT Inhibitor)	-
PI-103 (mTOR Inhibitor)	-
1PU (CDK 2 Inhibitor)	-
Palbociclib (CDK 4 Inhibitor)	-
Ribociclib (CDK 6 Inhibitor)	-
Letrozole (Aromatase Inhibitor)	-
Azacytidine (DNMT 1 Inhibitor)	-
Vorinostat (HDAC 1&2 Inhibitor)	-
Ciprofloxacin (Antibiotic)	F
